# Malaria-related perceptions and practices of women with children under the age of five years in rural Ethiopia

**DOI:** 10.1186/1471-2458-9-259

**Published:** 2009-07-23

**Authors:** Wakgari Deressa, Ahmed Ali

**Affiliations:** 1 School of Public Health, Addis Ababa University, P. O. Box 9086, Addis Ababa, Ethiopia

## Abstract

**Background:**

Malaria remains to be the major cause of morbidity and mortality among pregnant women and children in Ethiopia. The aim of this study was to investigate the local perceptions, practices and treatment seeking behaviour for malaria among women with children under the age of five years.

**Methods:**

This community-based study was conducted in 2003 in an area of seasonal malaria transmission in Adami Tulu District, south-central Ethiopia. Total samples of 2087 rural women with children less than five years of age from 18 rural *kebeles *(the smallest administrative units) were interviewed about their perceptions and practices regarding malaria. In addition, focus group discussions and in-depth interviews were conducted on similar issues to complement the quantitative data.

**Results:**

Malaria, locally known as *busaa*, is perceived as the main health problem in the study area. Mosquitoes are perceived to be the main cause of the disease, and other misperceptions were also widespread. The use of prevention measures was very low. Most mothers were familiar with the main signs and symptoms of mild malaria, and some of them indicated high grade fever, convulsions and mental confusion as a manifestation of severe malaria. Very few households (5.6%) possessed one or two nets. More than 60% of the mothers with recent episodes of malaria received initial treatment from non-public health facilities such as community health workers (CHWs) (40%) and private care providers (21%). Less than 40% of the reported malaria cases among women were treated by public health facilities.

**Conclusion:**

Malaria was perceived as the main health problem among women and children. The use of malaria preventive measures was low. A significant proportion of the respondents received initial malaria treatments from CHWs, private care providers and public health facilities. Concerted effort is needed to scale-up the distribution of insecticide-treated nets and improve the knowledge of the community about the link between malaria and mosquitoes. Effective antimalarial drugs should also be available at the grassroots level where the problem of malaria is rampant.

## Background

Despite the considerable increase in funds over the recent years to control malaria in Ethiopia, the disease has been the most frequently reported cause of morbidity and mortality [1]. Malaria transmission in the country is highly seasonal and often takes epidemic shape, with significantly higher malaria morbidity and mortality during the peak transmission season from September to December, following a heavy summer rainfall. All age groups are at risk of developing severe illness although children and pregnant women are biologically the most vulnerable.

Current malaria control interventions in Ethiopia include early diagnosis and prompt treatment with effective antimalarial drugs, preventive measures such as the use of insecticide-treated nets (ITNs) and indoor residual spraying (IRS), and malaria epidemic prevention and control [2,3]. However, similar to those countries in sub-Saharan Africa (SSA) with seasonal malaria transmission, intermittent preventive treatment (IPT) for malaria prevention during pregnancy has not been adopted in the country [4-7].

The Roll Back Malaria (RBM), Global Fund to Fight AIDS, Tuberculosis and Malaria (GFATM) and other global initiatives have recently improved the scale-up of anti-malaria interventions particularly for children less than five years of age and pregnant women. The main priority malaria control strategies among this group of population include early diagnosis and prompt treatment with effective antimalarials, and the use of preventive measures such as ITNs and IPT during pregnancy [8]. However, these interventions have not been effectively utilized by the target groups in Ethiopia due to accessibility and knowledge barriers.

In Ethiopia, artemether-lumefantrine replaced sulfadoxine-pyrimethamine (SP) in 2004 due to the increasing resistance of malaria to SP [9,10]. However, there has been a great concern about the availability and affordability of the newly introduced drug for community-based malaria control interventions carried out by village-based community health workers (CHWs) and home-based management of malaria [11-14]. The coverage and proper utilization of the most promising malaria preventive measure, ITNs, in the country is also limited by lack of sustainable distribution and issues related to replacement of free nets, seasonality of malaria, poor knowledge of the community with regard to the link between mosquitoes and malaria [15].

It is also well recognized that accessibility to anti-malaria interventions alone will not bring about the desired change. Several studies have demonstrated compliance to anti-malaria interventions depends substantially on social, behavioural and cultural factors that affect understanding of the causes, the relationship between mosquitoes and the disease, diagnosis, treatment and practices about prevention [16-19]. In addition, factors such as vulnerability, economic constraints, inadequacy or unavailability of appropriate health services, and other related factors play an important role in explaining health seeking behaviour of the people [20].

Studies in the past were mainly carried out in areas of stable malaria transmission due to the overwhelming burden of the disease among young children and pregnant women in those areas. Recently, however, there has been an increased need to understand perceptions and practices of the community about the disease, particularly health and treatment seeking behaviours in areas of seasonal transmission frequently exposed to malaria epidemics in SSA [21-24].

Due to the high burden of malaria among pregnant women and children under the age of five years, targeting women in the reproductive age group to deal with the disease has recently been widely recognized. It has been acknowledged that the success of malaria control in children and pregnant women depends on the understanding of the local socio-cultural factors affecting women's perceptions of causes and modes of transmission of the disease, health seeking behaviour and practices of malaria prevention measures [25-31]. However, research focusing on malaria issues among women in the reproductive age group with children under the age of five years in areas of seasonal transmission has particularly been scarce [21,27].

Therefore, understanding the local perceptions and practices of women is of utmost relevance to enhance community's potential to deal with village-based malaria interventions. This particular study was carried out in a rural community to examine women's perceptions and practices related to malaria transmission, symptoms/recognition, treatment, prevention, and treatment seeking behaviour in an area of seasonal malaria transmission. The purpose of the study was to generate information that might support malaria control program to design and implement appropriate interventions to control malaria. This study was part of studies that investigated maternal responses to childhood febrile illnesses and the associated socio-economic factors [32,33].

## Methods

### Study setting and population

The study was conducted in October–November 2003 in Adami Tulu District in south-central Ethiopia. The district lies at an altitude between 1500 m and 1600 m above sea level and covers an area of about 1403 km^2^. In 2002, the projected population of the district was 145 000 people with a density of 104/km^2^. The people are predominantly subsistence farmers belonging to the Oromo ethnic group and are primarily Muslims. The district is administratively organized into 62 rural and four urban *kebeles *(the lowest administrative units in the country).

Malaria transmission in the district is seasonal and epidemic type, peaking from September to December. Severe malaria epidemics occurred in the area in 1991, 1998 and 2003 [2,34,35]. Two species of human *Plasmodia *are present in the area; *Plasmodium falciparum *(about 70%) and *P. vivax *(about 30%). The district is characterized by widespread occurrence of drug resistance to chloroquine and SP. At the time of the study, therapeutic study of SP conducted between October and December 2003 at the district's malaria control laboratory (MCL) revealed 53.4% total treatment failure against *P. falciparum *[9]. The main malaria vector in the area was *Anopheles arabiensis *[36].

The health service coverage of the district was low; there were two health centers, three health stations, three health posts, one MCL, 34 community health workers (CHWs) and a number of private health care providers. At the time of the study, the first-line treatment for uncomplicated malaria at peripheral health facilities and community-based outlets such as CHWs was SP [37], and the implementation of ITNs for malaria prevention was at an early stage.

### Sample size estimation and sampling methods

The detail of the sample size calculation and sampling techniques was described else where [32,33]. In short, the sample was estimated at 2270 households with 30% expected prevalence of perceived malaria illness and 3.5% margin of error at 95% confidence level. Briefly, 18 of the 62 rural *kebeles *in the district were randomly selected, and all mothers/caretakers of children under five years of age were interviewed by house-to-house visits. Finally, the data for mothers with at least one child less than five years of age were separately analyzed and used for the preparation of this paper. For the qualitative research, the participants of the focus group discussions (FGDs) and in-depth interviews were purposively selected through discussions with community leaders and village coordinators.

### Data collection

Eighteen 10^th ^or 12^th ^grade graduate trained local interviewers administered structured questionnaires in the local language (i.e., *Afan Oromo*). The questionnaire was designed in English, translated into the local language and pre-tested before the actual use to validate its reliability. Information was collected on the socio-demographic characteristics of the mothers and their knowledge, perceptions and practices related to malaria transmission, symptoms, treatment and preventive methods. In addition, history of malaria morbidity during the previous three months and related practices of treatment seeking behaviour were assessed. Three supervisors who were experienced in field activities supervised the field work.

Three FGDs with an average of eight participants in each group and five in-depth interviews were held with mothers of children under the age of five years in three *kebeles *to further explore the modes of malaria transmission, signs and symptoms, health seeking behaviour and prevention about the disease. The qualitative data were used to supplement, cross check and further explore the quantitative findings. Open-ended FGD and interview guides originally prepared in English and translated into the local language were used for data collection by the first author with the assistance of two health workers from the District Health Office and village coordinators. In addition to field notes, each session was tape-recorded.

### Data analysis

All data collection forms were checked by field supervisors for completeness and reliability. The quantitative data were entered and cleaned using Epi Info software version 6.04d (CDC, Atlanta, GA, USA) and transferred to SPSS version 11 statistical software package (SPSS, Chicago, IL, USA) for analysis. Descriptive statistics such as frequencies and proportions were used for the analysis of the quantitative data. The FGDs and in-depth interviews from the different groups were transcribed and analyzed manually along the major themes of the study. Verbatim transcriptions in *Afan Oromo *were made for all tape-recorded FGDs and in-depth interviews, and finally translated into English. The findings from the individual interviews and FGDs were presented in an integrated manner. Where appropriate, quotes that best explained the perceptions and practices of the mothers about malaria were identified and used in parallel with the quantitative findings to elaborate more on the insights of the perceptions and practices of the community.

### Ethical considerations

This study was reviewed and approved by the Ethical Clearance Committee of the Faculty of Medicine at Addis Ababa University (AAU). Verbal informed consent was obtained from all respondents who participated in the study after explaining the purpose and objectives of the study in the local language. Participation in the study was voluntary and confidentiality of the information was assured both during and after data collection. The respondents were informed about their right either not to participate, not to answer any question or all of the questions. Any patient with symptoms suggestive of clinical malaria was treated with SP according to the national guidelines at the time of the study [37]. Patients with other illnesses were advised to seek medical care from appropriate service providers.

## Results

### Background characteristics of the mothers

A total of 2087 mothers with at least one child under the age of five years were interviewed from 3708 visited households in 18 *kebeles*. About 44% of the households did not have a woman with a child less than the age of 5 years. Of the total women with at least one child <5 years identified during the survey, almost all participated in the study, representing a response rate of 97.6%. A total of 24 and five women participated in three FGDs and five in-depth interviews, respectively.

Table 1 summarizes the background characteristics of the study mothers. Almost all (99.8%) were in the age group of 16–50 years, with a mean (± SD) age of 27 (± 6) years. A significant number of the mothers had no education (86.8%), and almost all (98%) were housewives. The average number of persons per household was 5.9, ranging from 2 to 15. The mean (± SD) number of living children per woman was 4 (± 2.3). The main source of income for most (98.6%) households was subsistence farming and livestock rearing. Almost all (99.1%) of the study households did not have a toilet facility at the time of the study. Firewood was the main type of fuel used for cooking in 89% of the surveyed households. The main source of drinking water included pond (43.7%), pipe water (28%) and river (19.5%). Firewood and water fetching in this community is exclusively conducted by the women and girls.

**Table 1:** Background characteristics of mothers, Adami Tulu District, Ethiopia, 2003

Characteristics (n = 2087)	N	%
Age in years		
15–24	639	30.6
25–34	1059	50.8
35–44	355	17.0
45–54	34	1.6
Marital status		
Currently married	2035	97.5
Widow	35	1.7
Divorced or separated	9	0.4
Never married (single)	8	0.4
Religion		
Muslim	1902	91.1
Orthodox Christian	132	6.3
Protestant Christian	38	1.8
Other	15	0.7
Ethnicity		
Oromo	1905	91.3
Siltie	111	5.3
Gurage	39	1.9
Amhara	12	0.6
Other	20	0.9
Educational status		
Illiterate	1812	86.8
Read only	30	1.4
Read and write	37	1.8
Elementary (grade 1–6)	196	9.4
Junior/secondary (grade 7–12)	12	0.6
Occupation		
Housewife	2045	98.0
Farmer	38	1.8
Other	4	0.1

### Perceived childhood health problems and their local terminology

We solicited information from women about the most common health problems of children in the area. Table 2 lists the *Afan Oromo *terms and their English equivalents for the common childhood illnesses reported by mothers. Malaria (99%), followed by diarrhea (56.7%) and eye diseases (34.3%), were the commonly reported childhood health problems. Mothers who reported malaria as a major health problem among children were further asked why they perceived malaria so, and 57% said that it was a common disease in the area, 54.5% reported that it was very severe and immediately kills a child, 51.5% replied that it mostly affected children, and 0.5% reported that it was incurable.

**Table 2:** Common health problems of children reported by mothers, Adami Tulu District, Ethiopia, 2003.

*Afan Oromo *term (n = 2087)	English equivalent	n (%)^a^
*Busaa*	Malaria	2066 (99.0)
*Garaa kaasaa*	Diarrhea	1183 (56.7)
*Dhukkuboota ijaa*	Eye diseases	715 (34.3)
*Gifira (Shiffee)*	Measles	516 (24.7)
*Dhukkubboota hanqina nyaataan dhufan*	Malnutrition	332 (15.9)
*Cittoo*	Scabies	98 (4.7)
*Afuura kutaa*	Shortness of breathing	95 (4.5)
*Raammoo garaa keessaa*	Intestinal parasitosis	87 (3.9)

^a ^Multiple responses possible.

The respondents used the local term *busaa *to refer to malaria. *Busaa *is literally translated as malaria, and we use this term in some instances in this paper when referring to malaria to emphasize the local term. *Busaa *was described as a term that included symptoms such as fever, shivering, chills, headache, back pain, loss of appetite, joint pain and vomiting. Almost all participants in all FGDs and in-depth interviews considered *busaa *as a serious disease that commonly affects children. No other disease was considered common in the area and more dangerous than malaria as illustrated in the expression stated below.

"The most common disease in this area is busaa. First, it makes the body cold, followed by headache and sometimes diarrhea. It is particularly common during this period (October–November) of the year". (FGD, Aneno village).

### Perceived symptoms and severity of malaria

Most mothers knew the symptoms of malaria. Overall, fever (97%), shivering and chills (94.2%), headache (72.1%) and back pain (60.8%) were considered as the most frequent symptoms associated with malaria. Other less frequently mentioned symptoms of malaria included loss of appetite (37.7%), body or joint pain (22.4%), vomiting (11.4%), and diarrhea (3.4%). Very few mothers (<1%) knew none of the symptoms of malaria. It was apparent that most mothers were able to recognize multiple symptoms of malaria, and the majority reported two or more symptoms, with 91.8% citing both fever and shivering/chills (Table 3). About 28% of the 2024 interviewees recognized fever, shivering/chills, headache, back pain and loss of appetite, while 24% reported fever, shivering/chills, headache and back pain, but didn't mention loss of appetite.

**Table 3:** Multiple symptoms of malaria as perceived by mothers, Adami Tulu District, Ethiopia, 2003.

Major malaria symptoms reported by mothers^a^					
	
Fever	Shivering and chills	Headache	Back pain	Loss of appetite	n (%)^b^
+	+	+	+	+	576 (28.0)
+	+	+	+	-	486 (24.0)
+	+	+	-	-	279 (14.0)
+	+	-	-	-	289 (14.3)
+	+	+	-	+	75 (3.7)
+	+	-	+	+	45 (2.2)
+	+	-	-	-	38 (1.9)

^*a *^"+" indicates the citation of the symptom, while "-" shows that the symptom is not reported by mothers.

^*b *^Percentage was calculated from 2024 mothers who reported fever

Similarly, fever, high body temperature, shivering, headache, thirsty, loss of appetite, vomiting and joint pain were reported in all FGDs as the main symptoms of malaria. The disease is recognized as malaria if one or more of these symptoms are manifested by a patient as illustrated below.

"If the body is hot, headache prevails, a patient is thirsty and drinks lot of water, back pain occurs, and when the body is chilly, we then recognize that the illness is due to malaria". (FGD, Aneno village).

Most of the participants in all FGDs identified two types of *busaa*: mild *busaa *and the severe type locally described as "*busaa isa sammuu nama koru (nama maraachu)"*. They narrated the latter as the type of malaria that quickly moves to the brain to make the patient unconscious (literally 'malaria of the brain'), and if left untreated, they said that one could either die or become mentally confused from it. Other participants also described this type of severe malaria as "*bicaa wobaa" *(a term borrowed from the *Amharic *word for yellow malaria). Signs and symptoms that were well articulated by participants of the FGDs and in-depth interviews for mild malaria included fever, headache, loss of appetite, thirst, joint pain and chills, whereas for those who presented with severe malaria included intense high-grade body fever (*qaama gubee ibidda namatti qabsiisa*), convulsion (*ni gaggabsa)*, and mental confusion (*sammuu koree nama maraacha*). In general, nearly all respondents and participants of FGDs and in-depth interviews perceived that malaria could kill a person within a short period if left untreated.

When the survey respondents were asked "Is malaria more serious for children or adults?", mothers (67.1%) thought that children were susceptible to contract malaria and develop severe illness, followed by a response that malaria was equally serious for both adults and children (29.3%). About 2% of the survey respondents, nevertheless, did not perceive malaria as a serious disease either to children or adults. In all FGDs and in-depth interviews it was recognized that *busaa *is an illness that affects all people, being more common among children and more severe to them. The effects of *busaa *during pregnancy were well articulated and understood by the participants, emphasizing the need for prompt actions. During the FGDs, although children and pregnant women were recognized as groups especially vulnerable to malaria, adults were also acknowledged to be at risk of the disease.

"Busaa affects both children and adults, but is more severe on children". It is serious and kills people. (In-depth interview, Abjata village).

### Knowledge of the cause for malaria

Most respondents (81%) said that malaria could be transmitted from one person to another. About 60% of the women perceived that malaria is transmitted by mosquitoes, followed by a response that incriminated sleeping together (38.7%) with a malaria patient as a cause for the disease. Other respondents also mentioned breathing from malaria patient (16.9%) and exposure to swampy areas and cold weather (4.9%) as a cause for malaria. Nearly 1% of the respondents who reported the transmissibility of malaria did not know how it is transmitted, while 9% said that either malaria could not be transmitted from one person to another or gave "did not know" responses.

The participants in all FGDs elaborated their ideas of the cause of malaria and most of them associated the disease with mosquito bites.

"There is a relationship between mosquito and malaria. When mosquito bites you, it transmits malaria to you". (FGD, Gerbi Gilgile village).

Some women in FGDs believed that malaria thrives during fasting when people do not get breakfast or food on time. Other mothers related the disease to the *Birraa *(relatively drier months from September to November) season of the year, during which food is surplus. They associated malaria with eating *eshet*. *Eshet *is young green maize, mainly available between September and November, usually eaten after frying it on fire, particularly in rural areas. It is during this time of the year when *eshet *is available that malaria is thought to be very common. Other misconceptions about the cause of malaria were also not uncommon.

"Malaria is caused by drinking dirty water on which mosquitoes breed". (In-Depth interview, Abjata village).

"Malaria is a major problem during Birraa season of the year. It is transmitted by mosquitoes and also by exposure to unhygienic areas. I think it is caused when mosquito bites you". (FGD, Abjata village).

The seasonality of malaria in the area was also described by the participants of the FGDs. Many perceived that malaria occurs primarily between September and December (i.e., *Birra *season). They argued that during this period, there is an increase in the number of mosquitoes. In the dry season, however, most of the participants indicated that the prevalence of the disease is low despite the fact that cases were found in the community.

### Knowledge and perceptions about malaria treatment

The best way of curing a person with malaria was explored; and 86.4% of the respondents reported the use of SP, 18.3% cited injections, 14.6% suggested the use of chloroquine, 3.7% indicated the use of herbal remedies and about 1% mentioned the importance of early treatment from health facilities. Very few (0.3%) respondents replied the use of religious healing such as holy water and *debtera *(i.e., local healer) for curing malaria. Less than 1% of women knew nothing about the best way to cure malaria.

With regard to the question whether malaria is a treatable disease or not, 98% replied "yes". Mothers who said that malaria is a treatable disease were further asked to list the names of the then available antimalarial drugs. About 95% replied SP, 45.6% reported chloroquine, and 5.5% mentioned paracetamol. Ninety-eight (4.8%) mothers were not able to mention the name of at least one antimalarial drug. About 93% of the mothers stated that SP was the most effective antimalarial for the treatment of malaria at the time of the survey.

Most respondents in the FGDs also mentioned the then used antimalarial drugs for the treatment of malaria. SP was the most commonly reported antimalarial drug and they knew that three tablets of it was the appropriate dose for adults. However, its inefficacy at the time of the survey was acknowledged among most participants in all FGDs and in-depth interviews and it was pointed out that malaria has become more difficult to treat unlike in the past. The following expression illustrates the perceived importance of SP in the treatment of malaria and the concern of people about its ineffectiveness.

"There is an antimalarial drug which is called "mecheresha" (local name for SP with a literal meaning of 'last resort and effective'). But I have a worry about the recent effectiveness of this drug. I think it is not working well now". (In-depth interview, Aneno village).

The use of herbal remedies in the treatment of malaria was also reported particularly in the remote areas of the district as described by one of the FGD participants. The use of garlic, ginger, and the leaves of local plants such as *hargisaa *and *irrettii *were reported to be common as a first response to malaria illness both among children and adults. The FGD participants elaborated that those substances were given orally to a patient with suspected malaria by grounding and mixing them with water. They believed that those substances were effective especially in reducing fever and vomiting, and if there could be no improvement, the next step identified was to take modern antimalarials at home or visiting the nearby health facilities. As one respondent in the FGD puts it:

"If a patient is given 'irrettii' [herbal remedy] at home, then it induces vomiting and diarrhea. As a result, the health of the patient would improve". (FGD, Abjata village).

### Knowledge and practices about malaria prevention

Mothers had several opinions about the preventability and ways to prevent malaria. About 94% of them believed that malaria could be prevented, while 5% hold the view that it could not. Few respondents (1.4%) did not know whether malaria could be prevented or not. It was reported by the respondents that they use a variety of methods to protect their family members from malaria. The proportion of mothers who reported their households' current use of different preventive methods against malaria and mosquito biting is summarized in Table 4.

**Table 4:** Percentage distribution of respondents citing different practices of malaria prevention methods by households, Adami Tulu District, Ethiopia, 2003

Method of malaria prevention (n = 2087)	N	%^a^
Drainage of mosquito breeding sites	931	44.6
Burn cow dung or leaves in the house	514	24.6
Blockage of mosquito entry holes	405	19.4
Use aerosol sprays	298	14.3
Closing doors and windows	219	10.5
Mechanical killing of mosquitoes	194	9.3
Use of mosquito net	117	5.6
Didn't use any protection method	694	33.3

^a ^Multiple responses possible.

Few households reported the use of preventive measures to protect themselves from malaria. Thirty-six percent of mothers reported having their home sprayed with DDT by government employed malaria control staff during the preceding summer (June-July). Most of the respondents in the FGDs believed that malaria could be prevented. The main preventive strategies mentioned by the group discussants included eliminating mosquito breeding sites and cleaning the environment around the compound.

Of the interviewed mothers, 40.1% (n = 837) said that they had heard of a mosquito net, and 78.4% said that its purpose was to kill mosquitoes, 65.9% replied that it protects against mosquito bite and 38.6% reported that it protects against malaria. About 2% did not know the purpose of mosquito net although they had heard about it. Only 14% (n = 117) of households with respondents who heard about a mosquito net owned ITNs. This indicates that only 5.6% of all the surveyed households with women respondents owned any type of mosquito net. Of those households that possessed nets, 93.2% owned one net, while 6.8% possessed two nets. The perceptions and practices of the respondents about mosquito net are illustrated in the expressions below.

"There is a material known as saaphana siree (local name for mosquito net). We were informed by the CHWs and health workers that, if properly used, it can prevent malaria. I know that some people in our village are currently using it". (FGD, Gerbi Gilgile village).

The FGD participants identified several factors that influence the possession and utilization of ITNs, and those included lack of confidence on mosquito net, high price of the net particularly for large families to acquire two or more nets, lack of understanding about the relationship between mosquitoes and malaria, poor understanding about the role of mosquito net in malaria prevention, lack of cultural use and the generally low awareness of the people about disease prevention and control. The presence of nets in the household was observed and confirmed by the interviewers. In households where there were ITNs, about 93% of the mothers reported sleeping under the nets the previous night, while 7% did not. However, investigation was not done on the current status of the nets, the re-treatment issues and how the nets were used.

### Mothers' experiences of malaria and treatment seeking behaviour

When the mothers were asked about their malaria history, 61.2% reported that they ever suffered from malaria. However, 4.1% of them reported that they had suffered from a febrile illness for which they were not sure about it. Of mothers who had ever suffered from malaria, 74.2% had also suffered from the disease within the preceding one year from the time of survey. However, due to the threat of recall bias, our analysis of the pattern of treatment seeking behaviour for malaria among them was limited to the most recent malaria episode self-reported within three months (n = 636) from the time of the survey, representing 30.5% of reported malaria prevalence among the study group over the stated period.

With regard to how their recent malaria illness was diagnosed, 65% recognized the illness themselves by the symptoms of the disease, 22.5% knew the disease by microscopic diagnosis and 8.8% knew the type of the disease from the health worker who examined them based on the signs and symptoms. Nineteen (3%) of them knew about their malaria illness after being diagnosed by CHWs and only 0.8% were told by a family member. Very few respondents (0.3%) were told by a traditional healer that they had malaria. In general, self-diagnosis was the primary diagnostic method for malaria in this rural community.

**Figure 1:**
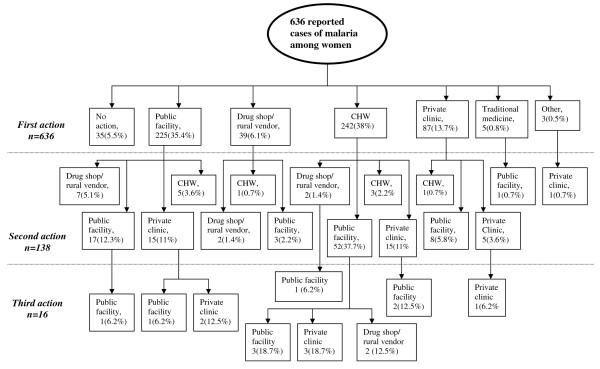
**Malaria treatment choices for women who reported recent illness**. Public facility includes health center, health station, health post, and hospital and malaria control laboratory. CHW: Community Health Worker. Adami Tulu District, Ethiopia, 2003.

For the question "How many times/different places did you seek treatment for the recent episode of malaria?": 5.5% (n = 35) did nothing, 72.8% (n = 463) reported one source of treatment, 19.2% (n = 122) stated two places and 2.5% (n = 16) reported three sources of treatment. Table 5 summarizes the patterns of visits to various care providers in three attempts among those who did something or sought treatment (n = 601), excluding 5.5% of those who did not take any action. An additional visit in Table 5 indicates that recovery was not successful in the previous visit. Among patients who made the first visit to various sources of antimalarial treatment, about 77% (463 of 601) reported recovery from the illness. About 23% (n = 138) of those who visited the first source of antimalarial treatment made a subsequent visit to the second source of treatment, of whom 12% again resorted to the third choice of treatment. The detail of the patterns of resort for treatment seeking for malaria in the study women is presented in Figure 1. The patterns highlighted in this study indicate that multiple visits were more common among those women who made the first visit to CHWs.

**Table 5:** Women's pattern of visits to sources of antimalarial treatment, Adami Tulu District, Ethiopia, 2003

Source of treatment	First visit, n (%)	Second visit, n (%)	Third visit, n (%)
CHWs^a^	242 (40.3)	10 (7.2)	0 (0.0)
Public health facility	225 (37.4)	81 (58.7)	8 (50.0)
Private clinic	87 (14.5)	36 (26.1)	6 (37.5)
Drug shop/vendor	39 (6.5)	11 (8.0)	2 (12.5)
Traditional medicine	5 (0.8)	0 (0.0)	0 (0.0)
Other sort	3 (0.5)	0 (0.0)	0 (0.0)

	601 (100)	138 (100)	16 (100)

^a ^Community Health Worker.

Most of the women participants in all FGDs and in-depth interviews indicated similar patterns of treatment seeking behaviour for malaria. CHWs, public facilities and private clinics were identified as the main sources of treatment for malaria. In all FGDs, the inefficiencies of public health facilities mainly due to long waiting time, overcrowding, health workers misconduct and drug prescription without being examined in the laboratory were the main deterrents reported. However, private care providers were perceived to be rendering better quality particularly due to the availability of drugs and injections, but incriminated for expensive services. On the other hand, proximity to the village, free antimalarial treatment and good interpersonal relationship and communication with CHWs made their services to be more utilized by patients with perceived malaria.

## Discussion

The study women have an understanding of malaria aetiology, symptoms, severity, treatment and preventive methods, as observed in other study areas [17,19,26,38,39]. The local term *busaa *is shared by all members of the community and considered to be a common and widespread health problem affecting both children and adults, particularly during the peak transmission season from September to December, a time that overlaps with the major harvest in the area. Although the malaria situation in the area is seasonal, about 60% of the respondents perceived the role of mosquito bite in malaria transmission, showing similarity with the findings from intense transmission areas in SSA [17,26,39].

Although mosquitoes were recognized to be the main agents in the transmission of malaria in this study, misperceptions such as eating *eshet*, sleeping together with a malaria patient, hunger and exposure to unhygienic surroundings were widely perceived as a cause of malaria. These misconceptions were also common in those respondents who recognized the role of mosquito in the chain of malaria transmission. In Ghana, it has been reported that malaria is presumed to be caused as a result of excessive heat and eating oily or starchy food [17,19,38]. In Uganda, malaria is believed to be caused by poor diet and exposure to bad environmental conditions [26]. In Guatemala, malaria is thought to be caused by exposure to cold or wet conditions, weakness or poor general health, poor eating habits and problems related to hygiene [39]. A study from coastal Kenya also found that most mothers did not know the association between mosquitoes and malaria [40]. People's perceptions and understandings about the perceived cause and transmission of malaria have strong implications on the preventive measures such as the current scale-up ITNs implementation [17,19].

The majority of the study participants recognized two or more symptoms of malaria. This is of obvious importance in a home-based management of malaria among children under the age of five years. The high level of knowledge pertaining to severe malaria was striking. However, there is definitely a need for further exploration of mother's views about causes and health seeking behavior for managing convulsions in children due to malaria. Many studies have identified a strong belief that convulsions are due to supernatural causes and best treated by traditional medicine [22,29,40,41].

Despite the high level of general knowledge on malaria, the use of appropriate malaria preventive measures was low in our study setting. Although draining of mosquito breeding sites, cleaning the environment, smoking, blockage of mosquito entry holes and aerosol sprays were reported as common practices of households to prevent malaria, the practice was not observed and might be influenced by social desirability bias in which the respondents replied to conform to the expectations of the health workers.

Very few households in the study area possessed mosquito nets during the assessment since that measure was a newly adopted control strategy in Ethiopia. The practice of malaria prevention by households is related to perception of the risk, their knowledge of the causes of malaria and its prevention measures [19]. A recent study from an area of seasonal malaria transmission in Eritrea indicates that correct knowledge of malaria transmission through mosquito bite was found to be a good predictor of ITN possession and use within households [42]. Studies have also demonstrated a link between the demand for ITNs and the knowledge of the benefits of the nets [17,19].

The present study was conducted prior to the massive scale-up of ITNs in malaria endemic areas of Ethiopia. Since 2005, important steps have been under taken particularly to scale-up the implementation of ITNs in the country. A household cluster survey conducted in Oromia and Southern Nations, Nationalities and People's Region (SNNPR) by the Carter Center in January 2007 revealed that 45.4% of the surveyed households in Oromia and 51.2% in SNNPR owned at least one mosquito net of any type and the coverage for at least one long-lasting insecticidal nets (LLINs) was 32.5% and 40.1%, respectively [43], indicating a ten-fold increase compared to the Demographic and Health Survey 2005 results of less than 1% in the two regions [44]. The National Malaria Indicator Survey carried out from October to December 2007 indicates that in areas below 2000 m, about 65% of the households owned at least one LLIN [45].

Despite the study participant's higher knowledge of malaria, their low educational level implies the need for intensifying the expansion of schools for raising the level of women's education. Evidences show that educational attainment is associated with better malaria knowledge. For example, in Nigeria, higher levels of education were associated with improved knowledge and practice about the appropriate malaria prevention and control interventions [46]. Knowledge of malaria was found to be positively associated with level of education in Zambia; however, this knowledge did not translate into increased mosquito net use [47]. In Uganda, malaria knowledge was found to be related to net ownership [48]. In Nigeria, a study found a statistically significant higher knowledge of malaria among mothers who were educated, skilled or professionals than among the uneducated or unskilled category [49]. Education also increases the probability that households would purchase ITNs [46]. This suggests that higher level of education may be required to impact upon the intake of malaria prevention and control interventions.

We found that people with malaria get treatment from a variety of sources, as elsewhere in SSA [50]. In this seasonal transmission area, the first response to febrile illnesses suspected to be malaria was seeking care from outside the home mainly from CHWs, public and private health facilities. Nearly 50% of the mothers first sought treatment for malaria from a health facility, suggesting that about half of the people with malaria do not seek care from the public health sector. The most common reasons for utilizing CHWs were greater proximity and free treatment.

Home treatment and the use of herbal/traditional remedies were found to be very low in this study. In a study of treatment seeking behaviour among women in Uganda, the use of local herbs and self-treatment were found to be the first resort for malaria treatment, followed by seeking treatment from formal care [26]. Most studies across Africa in areas of stable malaria transmission indicate that the first response to malaria illness is home treatment [26,40,50-52]. The use of health care facilities as the first response for malaria treatment was reported in limited studies. In Uganda, more than 60% of those who had experienced an episode of malaria during an epidemic first sought treatment from a health facility [53]. In addition, in one study in an area of seasonal malaria transmission in Kenya, health facilities were initially used by 26% of persons with fever, with the majority first visiting the informal retail sector [23]. The main factors influencing initial treatment seeking from health facilities include severity of the illness, cost of treatment, poor access to health facilities, long waiting time, dissatisfaction with the performance and behaviour of health workers, and unavailability of appropriate drugs [50].

The findings of this study show that SP is well accepted as a treatment for perceived malaria. However, people seem to be concerned with its efficacy. The dissatisfaction of the community about the efficacy of SP is inline with the nationwide failure of SP for the treatment of falciparum malaria in Ethiopia in 2003 [9]. In 2004, Ethiopia adopted a new antimalarial drug policy and abandoned SP as the first-line treatment for confirmed uncomplicated falciparum malaria in favour of ACT (artemether-lumefantrine) [10,54]. The decision created the need to find ways of availing and effectively administering the new drug. The consequences of the lack of effective and affordable antimalarial drugs are clear. SP-resistant malaria has spread virtually throughout SSA and many countries have changed their first-line drug to ACTs [13].

At present, there are several caveats with regard to the treatment of malaria at community level. In the recent past, using SP at community level through home management of malaria strategy raised hope to reduce the burden of malaria morbidity and mortality particularly among <5 children. Ethiopia adopted home management of malaria strategy using trained mother coordinators and CHWs [2] based on the promising findings from the randomized clinical trial study conducted in the northern part of the country using trained mother coordinators to provide early treatment of malaria for children using chloroquine [55]. However, the implementation of this strategy has recently been compromised due to lack of an effective and cheap drug that can be easily administered at community level [3,10]. It is hoped that the current deployment of health extension workers in the country would result in improved anti-malaria interventions such as ITNs and ACTs at the grassroots level.

Like all questionnaire-based cross-sectional studies, this study is limited by the self-reported data, which are susceptible to recall and information biases. We attempted to minimize those biases through piloting the questionnaire and training of data collectors and field supervisors. The other limitation of the study is that malaria was assessed by the local term "*busaa*" where symptoms such as fever, headache, and vomiting might as well have been due to other illnesses, resulting in misclassification problems. However, most malaria cases in Ethiopia are treated based on the signs and symptoms of the disease. Therefore, the significance of our study might be substantial as the study was conducted during peak malaria transmission season.

## Conclusion

The findings of this study indicate the importance of understanding women's perspective in community-based malaria prevention and control. The envisaged reduction in malaria morbidity and mortality will depend on the successful implementation of malaria control strategies by involving the local community. A more concerted effort is needed for scaling-up the distribution of ITNs, improving the knowledge of the community about the link between malaria and mosquitoes, causation of malaria and its preventive methods particularly on the proper utilization of ITNs. One important area that must be given priority in the effective implementation of ITNs is considering the pivotal role of women in the community. Women are role models for their family members particularly for their children. Raising their awareness and understanding, and involving them in malaria prevention and control could enhance the proper use of ITNs by their children as well as other family members. Effective antimalarial drugs should also be available at the grassroots level where the problem of malaria is highly widespread.

## Competing interests

The authors declare that they have no competing interests.

## Authors' contributions

WD was responsible for the conception, study design, implementation, data analysis, interpretation and for the preparation of the draft manuscript. AA involved in the design, contributed to the writing, interpretation and critical revision of the paper for intellectual content.

## Pre-publication history

The pre-publication history for this paper can be accessed here:


